# Sulforaphane rescues amyloid-β peptide-mediated decrease in MerTK expression through its anti-inflammatory effect in human THP-1 macrophages

**DOI:** 10.1186/s12974-018-1112-x

**Published:** 2018-03-12

**Authors:** Kyoung A. Jhang, Jin-Sun Park, Hee-Sun Kim, Young Hae Chong

**Affiliations:** 1 Department of Microbiology, Division of Molecular Biology and Neuroscience, School of Medicine, Ewha Medical Research Institute, Ewha Womans University, Seoul, 158-710 Republic of Korea; 2 Department of Molecular Medicine, Tissue Injury Defense Research Center, School of Medicine, Ewha Womans University, Seoul, 158-710 Republic of Korea

**Keywords:** Alzheimer’s disease, Aβ1-42, MerTK, Sulforaphane, Innate immune response

## Abstract

**Background:**

Mer tyrosine kinase (MerTK) activity necessary for amyloid-stimulated phagocytosis strongly implicates that MerTK dysregulation might contribute to chronic inflammation implicated in Alzheimer’s disease (AD) pathology. However, the precise mechanism involved in the regulation of MerTK expression by amyloid-β (Aβ) in proinflammatory environment has not yet been ascertained.

**Methods:**

The objective of this study was to determine the underlying mechanism involved in Aβ-mediated decrease in MerTK expression through Aβ-mediated regulation of MerTK expression and its modulation by sulforaphane in human THP-1 macrophages challenged with Aβ1-42. We used protein preparation, Ca^2+^ influx fluorescence imaging, nuclear fractionation, Western blotting techniques, and small interfering RNA (siRNA) knockdown to perform our study.

**Results:**

Aβ1-42 elicited a marked decrease in MerTK expression along with increased intracellular Ca^2+^ level and induction of proinflammatory cytokines such as IL-1β and TNF-α. Ionomycin A and thapsigargin also increased intracellular Ca^2+^ levels and production of IL-1β and TNF-α, mimicking the effect of Aβ1-42. In contrast, the Aβ1-42-evoked responses were attenuated by depletion of Ca^2+^ with ethylene glycol tetraacetic acid. Furthermore, recombinant IL-1β or TNF-α elicited a decrease in MerTK expression. However, immunodepletion of IL-1β or TNF-α with neutralizing antibodies significantly inhibited Aβ1-42-mediated downregulation of MerTK expression. Notably, sulforaphane treatment potently inhibited Aβ1-42-induced intracellular Ca^2+^ level and rescued the decrease in MerTK expression by blocking nuclear factor-κB (NF-κB) nuclear translocation, thereby decreasing IL-1β and TNF-α production upon Aβ1-42 stimulation. Such adverse effects of sulforaphane were replicated by BAY 11-7082, a NF-κB inhibitor. Moreover, sulforaphane’s anti-inflammatory effects on Aβ1-42-induced production of IL-1β and TNF-α were significantly diminished by siRNA-mediated knockdown of MerTK, confirming a critical role of MerTK in suppressing Aβ1-42-induced innate immune response.

**Conclusion:**

These findings implicate that targeting of MerTK with phytochemical sulforaphane as a mechanism for preventing Aβ1-42-induced neuroinflammation has potential to be applied in AD therapeutics.

## Background

Alzheimer’s disease (AD), a progressive age-related disease, is the most common neurodegenerative disorder primarily affecting the elderly population over the age of 60 years. Amyloid-β (Aβ) deposition, neurofibrillary tangle formation, and neuroinflammation are major pathogenic mechanisms that lead to neocortical and hippocampal atrophy, memory dysfunction, and cognition decline in AD patients [1]. Chronic neuroinflammation including reactive astrocytes, activated microglia, and enhanced cytokine load elicited by the accumulation and subsequent deposition of Aβ within the brain plays a critical role in the initiation and progression of AD [2, 3]. Its important role in AD has been highlighted by a succession of genetic studies identifying numerous immune mediators such as triggering receptor expressed on myeloid cells (TREM) and others linked to elevated AD risk [4, 5]. Up to date, available therapeutic agents are only able to slow down the progression of AD with limited benefits.

Tyro3, Axl, and Mer tyrosine kinase (MerTK) (TAM) family receptor tyrosine kinases (RTKs) together with their ligands Gas6 and PROS1 are negative feedback regulators that can reduce inflammation in tissue macrophages [6, 7]. Aberrant expression and dysregulated activation of TAM family members have demonstrated that TAM receptors are both controllers of microglial physiology and potential targets for therapeutic intervention for a variety of central nervous system (CNS)-related disorders, including AD [8, 9]. In fact, earlier report has described that Gas6, a ligand of TAM receptors, has a protective role in Aβ-induced apoptosis [10]. Tyro3 overexpression in HEK293 cells stably expressing a mutant amyloid precursor protein has been linked to decreased Aβ accumulation while Tyro3 partial knockdown in vivo is associated with increased amyloid plaque formation [11]. Besides, TAM receptors play pivotal roles in adult hippocampal neurogenesis. The loss of these receptors can cause comprised neurogenesis in the dentate gyrus of adult hippocampus [12]. Notably, a recent study has demonstrated that induction of MerTK expression in plaque-associated macrophages consequently licensed their phagocytic activity and promoted plaque clearance in murine models of AD [13]. This strongly suggests that Mer receptors play a vital role in rapid reduction of plaque burden. In addition, MerTK activity necessary for amyloid-stimulated phagocytosis strongly implicates that MerTK dysregulation might contribute to chronic inflammation indicated in AD pathology. However, the precise mechanism involved in the regulation of MerTK expression by Aβ1-42 in proinflammatory environment has not yet been ascertained.

Among naturally occurring dietary phytochemicals, isothiocyanate sulforaphane derived from cruciferous vegetables such as broccoli has received considerable attention as an alternative candidate for AD therapy due to its safety, efficacy, and blood–brain barrier penetration [14]. Indeed, sulforaphane can ameliorate the cognitive function of Aβ-induced AD acute mouse models [15] and decreased locomotor activity in mice with AD-like lesions [16]. Moreover, sulforaphane protects the brain from Aβ-induced oxidative cell death via activating nuclear factor erythroid 2-related factor 2 (NRF2) signaling cascade [17] which induces cytoprotective proteins including heme oxygenase-1 in the CNS [14]. Recently, we have reported that sulforaphane possesses anti-inflammatory activity against Aβ peptide via signal transducer and activator of transcription-1 (STAT-1) dephosphorylation and activation of NRF2/HO-1 cascade in human THP-1 macrophages [18]. Nonetheless, direct evidence indicating that sulforaphane can regulate Aβ1-42-induced effect on MerTK expression during inflammatory responses has not been reported. In addition, the potential mechanism of sulforaphane involved in the modulation of MerTK expression in human microglia-like THP-1 cells is currently unclear.

Therefore, the objective of this study was to determine the underlying mechanism involved in the Aβ-mediated regulation of MerTK expression and its modulation by sulforaphane in human THP-1 macrophages in vitro. Results of the present study indicated that Aβ1-42 could downregulate the level of MerTK protein via increasing intracellular Ca^2+^ level and NF-κB activation, thereby overproducing interleukin 1β (IL-1β) and tumor necrosis factor-α (TNF-α), which could act as negative feedback regulators of MerTK expression. Notably, these effects of Aβ1-42 can be significantly reversed by sulforaphane in human THP-1 macrophages. Moreover, small interfering RNA (siRNA)-mediated knockdown of MerTK diminished sulforaphane’s anti-inflammatory effect on Aβ1-42-mediated induction of IL-1β and TNF-α, implicating a critical role of MerTK in the negative regulation of Aβ1-42-induced innate immune response. Collectively, these findings implicate that targeting of MerTK with phytochemical sulforaphane as a mechanism for preventing Aβ1-42-induced neuroinflammation has potential to be applied in AD treatment strategies.

## Methods

### Materials

Synthetic siRNA for MerTK and nonspecific control siRNA were purchased from Santa Cruz Biotechnology (Santa Cruz, CA, USA). Lipofectamine 2000 was purchased from Invitrogen (Carlsbad, CA, USA). Antibodies against lamin B1 and MerTK were obtained from Abcam (Cambridge, UK). Anti-NF-κB was obtained from Cell Signaling Technology (Danvers, MA, USA). Sandwich enzyme-linked immunosorbent assay (ELISA) kits for human TNF-α and IL-1β were purchased from BD Biosciences (San Diego, CA, USA). Horseradish peroxidase-conjugated anti-mouse IgG and anti-rabbit IgG were obtained from Jackson ImmunoResearch (West Grove, PA, USA). Sulforaphane and BAY 11-7082 were also acquired from Abcam. Pre-immune IgG was obtained from BioLegend (San Diego, CA, USA). Recombinant proteins of human TNF-α, IL-1β, and monocyte chemoattractant protein-1 (MCP-1) were purchased from R&D Systems (Minneapolis, MN, USA). Actinomycin D (inhibitor of de novo mRNA expression) and cycloheximide (inhibitor of protein synthesis) were obtained from Calbiochem (La Jolla, CA, USA). Ionomycin (Ca^2+^ ionophore) and thapsigargin (an endoplasmic reticulum Ca^2+^ pump inhibitor) were acquired from Sigma-Aldrich (St. Louis, MO, USA). Anti-β-actin antibody and other chemicals were also acquired from Sigma-Aldrich.

### Preparation of Aβ peptides

Aβ1-42 peptide was purchased from American Peptides (Sunnyvale, CA, USA) and prepared before use as described previously [19, 20]. Aβ1-42 peptide was dissolved in dimethyl sulfoxide at 5 μM to be diluted to 250 μM in double-distilled water before experiments. This preparation mostly contained a monomeric form of Aβ1-42 with very small amounts of dimers and larger oligomers up to 6-mers [21].

### Differentiation of human microglia-like THP-1 cells

Human monocytic cell line THP-1 was obtained from American Type Culture Collection (ATCC, Rockville, MD, USA) and maintained in RPMI 1640 containing 10% heat-inactivated fetal calf serum as described previously [21, 22]. THP-1 has been widely used as a model of human monocytes/macrophages or microglia due to its functional and morphological similarities, including its capacity for signal transduction pathways as well as its functional differences in distinct species [22]. Human monocyte-derived macrophages share many phenotypic and functional features with human microglial cells (so-called brain macrophages). Thus, all experiments required THP-1 cells to be differentiated to explore substantial changes in responsiveness during differentiation from monocyte to macrophage. THP-1 cells (10^5^ cells/mL) were seeded into 96-well culture plates and incubated with 20 nM phorbol 12-myristate 13-acetate (PMA) for 48 h to become adherent to plastic culture dish and develop morphology of differentiated macrophages most closely resembling microglia as described previously [21, 22].

### Experimental treatment

After washing, adherent THP-1 macrophages were incubated at 37 °C with serum-free RPMI supplemented with 0.5% glucose for 1 h before stimulation by adding Aβ1-42 peptide in the presence or absence of sulforaphane (5 μM). In some experiments, cells were incubated with ionomycin A or thapsigargin to determine the effect of increased intracellular Ca^2+^ level. To examine the relationship between proinflammatory cytokines and MerTK expression, THP-1 cells were also exposed to recombinant TNF-α, IL-1β, or MCP-1. All concentrations were selected based on the maximal effect of the drug on its specified target. Vehicles were treated identically without Aβ1-42 or pharmacological agents. After stimulation with Aβ1-42 and/or specific agent for an appropriate time, total cell lysates and supernatants were prepared and stored at − 20 °C until use for Western blot analysis as described below. Concentrations of human IL-1β or TNF-α in culture media were also analyzed as described below.

### Calcium imaging and fluorescence measurements

To visualize intracellular steady-state Ca^2+^ levels, THP-1 cells were stained by adding Fluo-3A in its acetoxymethyl ester form (Fluo-3AM) to culture media at a final concentration of 5 μg/mL throughout Aβ1-42 or vehicle treatment as described previously [20]. Ca^2+^ influx fluorescence images were captured after the indicated treatment. Images were recorded using an inverted microscope (Nikon Eclipse TE300) and analyzed with ImageJ program. An increase of intracellular Ca^2+^ level in different cultures was expressed in fold compared to that in vehicle-treated control for each individual experiment.

### Nuclear fractionation

Cells were harvested and lysed on ice in 100 μL of lysis buffer A (10 mM Tris-HEPES (pH 7.9), 10 mM KCl, 1.5 mM MgCl_2_, 0.1 mM EDTA, 0.5 mM dithiothreitol, 0.5 mM phenylmethylsulfonyl fluoride, and 1% NP-40) for 4 min as described previously [19]. After centrifugation at 3000*g* for 10 min, cell pellet was resuspended in 50 μL of extraction buffer B (20 mM HEPES (pH 7.9), 20% glycerol, 1.5 mM MgCl_2_, 1 mM EDTA, 0.5 mM dithiothreitol, and 0.5 mM phenylmethylsulfonyl fluoride), incubated on ice for 30 min, and centrifuged at 13,000*g* for 5 min. Nuclear proteins were stored at − 70 °C after determining protein concentration. Nuclear fractions were then subjected to Western blot analysis.

### siRNA studies

Transfection of cells with siRNA was performed using Lipofectamine^®^ 2000 transfection reagent as described previously [19, 21]. Commercially available human MerTK and negative control siRNA were used for transfection at indicated concentrations. Briefly, at 16 h after transfection, cells were treated with sulforaphane for 30 min prior to treatment with Aβ1-42 for 16 h. Levels of IL-1β or TNF-α in culture supernatant were analyzed using human-specific IL-1β or TNF-α ELISA kit (BD Biosciences).

### Electrophoresis and Western blotting

Immunoblotting was conducted as described previously [19, 20]. Briefly, equal quantities of sample proteins were subjected to 11% sodium dodecyl sulfate-polyacrylamide gel electrophoresis (SDS-PAGE), transferred to polyvinylidene difluoride membranes (GE Healthcare, Buckinghamshire, UK), blocked with 3% milk in Tris-buffered saline-Tween for 0.5 h, and probed with primary antibody diluted with 1% milk and incubated at 4 °C overnight. After incubating with horseradish peroxidase-conjugated secondary antibodies (Jackson ImmunoResearch), signals were acquired with an enhanced chemiluminescence system. Densitometric values were normalized against levels of β-actin.

### ELISA

Differentiated THP-1 cells were treated with a variety of stimuli as indicated, and concentrations of human IL-1β or TNF-α in culture media were evaluated with sandwich ELISA kits (BD Biosciences) in accordance with the manufacturer’s recommendations. Standard curves were obtained using recombinant human IL-1β or TNF-α.

### Statistical analyses

Differences between groups were evaluated for statistical significance using one-way ANOVA and Student’s *t* test. Null hypotheses of no difference were rejected if *p* value was less than 0.05.

## Results

### Aβ1-42 treatment reduces MerTK expression in human THP-1 macrophages

**Fig. 1:**
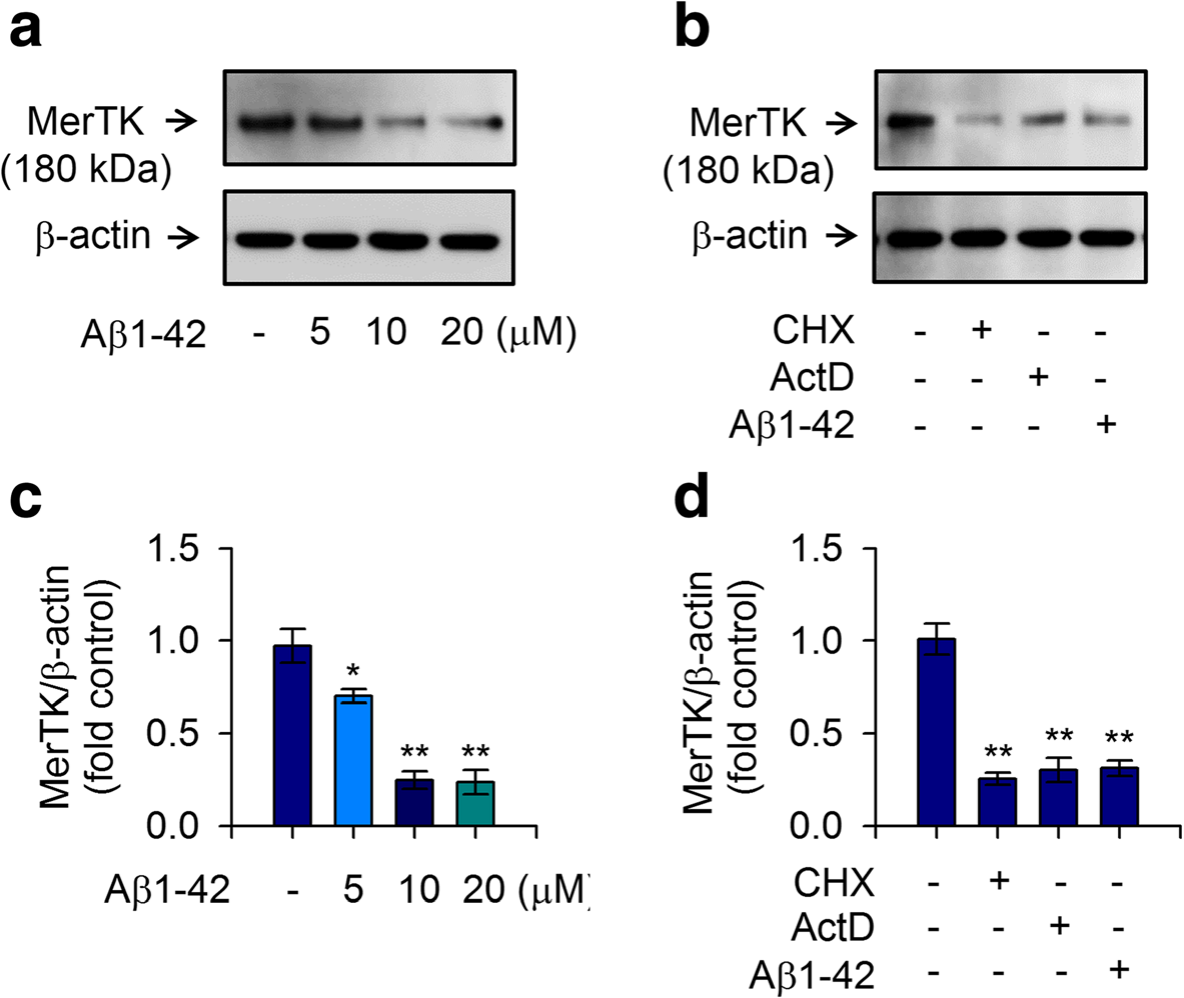
MerTK expression in human THP-1 macrophages is decreased by treatment with Aβ1-42. To measure MerTK expression in response to Aβ1-42 stimulation, THP-1 cells were incubated with either the vehicle only (−) or increasing amounts of Aβ1-42 for 8 h in serum-free RPMI 1640 medium supplemented with glucose (0.5%). **a** Total cell lysates were examined for MerTK protein via immunoblot. Aβ1-42 decreased MerTK in a dose-dependent manner. **b** THP-1 cells were also incubated with cycloheximide (CHX, 2 μM), actinomycin D (ActD, 100 nM), or Aβ1-42 (10 μM) for 8 h. Western blot analyses were conducted to determine the effect of actinomycin D or cycloheximide on MerTK expression. Levels of β-actin were examined to ensure equal amounts of total protein as a loading control. Results are representative of three independent experiments. **c**, **d** Quantitative analyses of **a** and **b** showing levels of MerTK protein. All data are presented as means ± SEM (*n* = 3). *P < 0.05, **P < 0.01, versus vehicle-treated samples. ActD, actinomycin; Aβ, amyloid-β; CHX, cyclohexamide, MerTK, Mer tyrosine kinase

To clarify the pathological mechanism related to MerTK in AD, we measured the expression level of MerTK in response to stimulation by Aβ1-42 in human THP-1 macrophages. We treated cultured THP-1 macrophages with Aβ1-42 for 16 h and found that treated THP-1 cells expressed lower levels of MerTK protein than naive cells in a dose-dependent manner. Significant reduction in MerTK protein level was found after treatment with 5 μM of Aβ1-42. MerTK protein level was further decreased after treatment with 10 μM Aβ1-42 (Fig. 1a, c). Since a very similar level of reduction in MerTK protein level was obtained after treatment with 20 μM of Aβ1-42 compared to that after treatment with 10 μM of Aβ1-42, the lower concentration (10 μM) of Aβ1-42 was used for the following experiments. Notably, MerTK protein was consistently reduced when de novo mRNA expression and protein synthesis were inhibited by actinomycin D and cycloheximide, respectively (Fig. 1b, d). Our data confirmed that Aβ1-42 elicited a significant decrease of MerTK expression in human THP-1 macrophages in a dose-dependent manner and the reduction of MerTK expression was at both transcriptional and translational levels.

### An increase of intracellular Ca^2+^ level upon Aβ1-42 stimulation is responsible for the reduction in MerTK expression

**Fig. 2:**
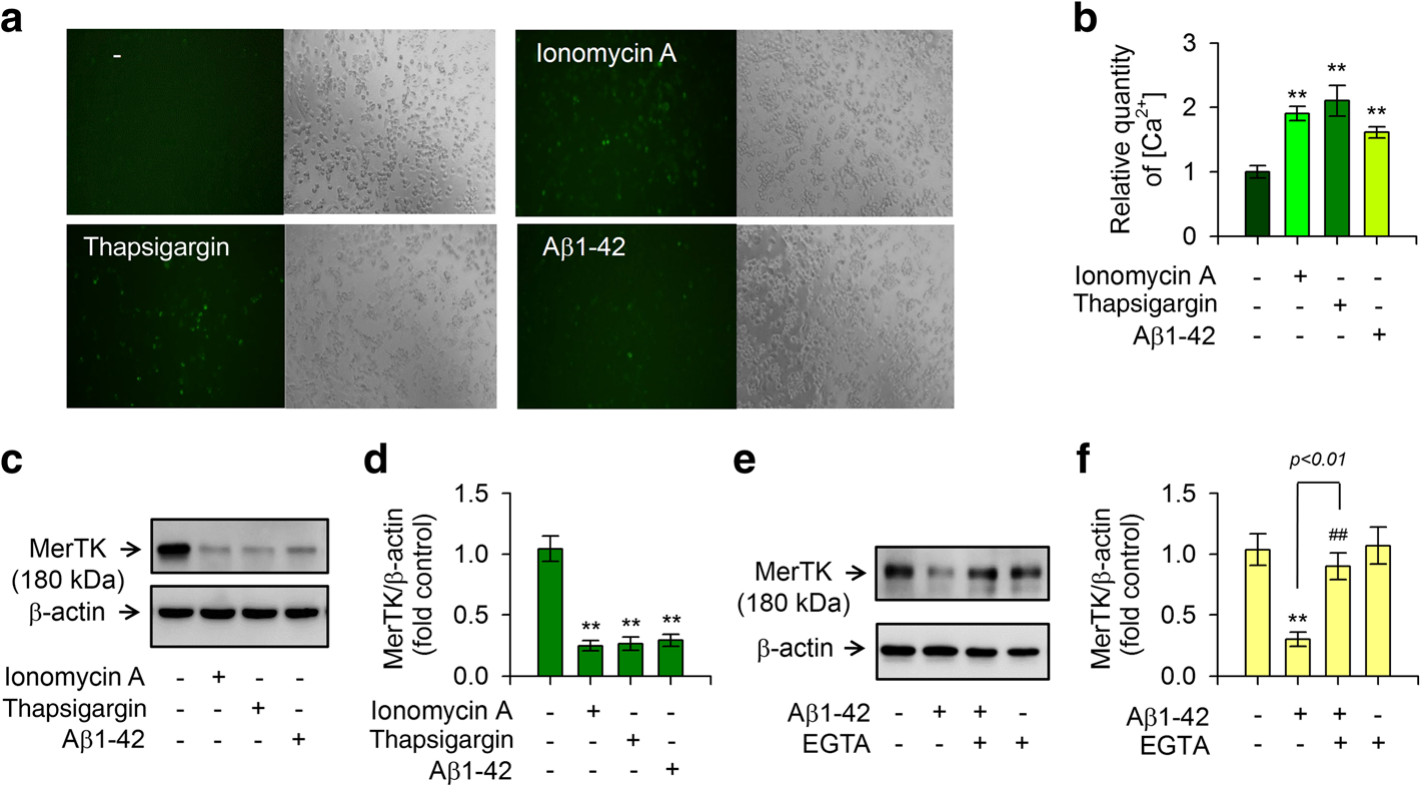
Aβ1-42-mediated reduction of MerTK expression is associated with increased intracellular Ca^2+^ level compared to treatment with ionomycin A or thapsigargin. **a** Intracellular Ca^2+^ images were obtained by Fluo-3AM method at 8 h after treatment with either the vehicle only (−), ionomycin A (4 μM), thapsigargin (2 μM), or 10 μM Aβ1-42 in THP-1 macrophages. **b** Histogram showing the ratio of intracellular Ca^2+^ levels to vehicle-treated group. **c** THP-1 cells were incubated with either the vehicle only (−), ionomycin A (4 μM), thapsigargin (2 μM), or Aβ1-42 (10 μM) for 8 h. **d** Densitometric quantification of analyses of **c** showing levels of MerTK protein. **e** THP-1 cells were pretreated with EGTA (0.5 mM) for 30 min followed by incubation with either the vehicle only (−) or Aβ1-42 (10 μM) for 8 h. Total cell lysates were examined for MerTK via immunoblot. MerTK expression was decreased when intracellular Ca^2+^ levels were increased by ionomycin A, thapsigargin, or Aβ1-42. In contrast, the Aβ1-42-mediated decrease of MerTK protein was attenuated by depletion of Ca^2+^ with EGTA. Results are representative of three independent experiments. **f** Densitometric quantification of analyses of **e** showing levels of MerTK protein. All data are presented as means ± SEM (*n* = 3). **P < 0.01, versus vehicle-treated samples; ^##^*P* < 0.01, compared to Aβ1-42-treated samples. Aβ, amyloid-β; EGTA, ethylene glycol trtraacetic acid; MerTK, Mer tyrosine kinase

The increase of intracellular Ca^2+^ level may initiate inflammatory response in activated microglia. We have observed that Aβ1-42 can extensively increase intracellular levels of Ca^2+^ in murine microglial BV2 cells [23] and THP-1 monocytes [20] using the Fluo-3AM method. Thus, we investigated the role of intracellular Ca^2+^ level in the reduction of MerTK protein level elicited by Aβ1-42 insult. Our results showed that 10 μM Aβ1-42 significantly increased intracellular Ca^2+^ levels in THP-1 macrophages using the Fluo-3AM method despite the low magnitude of intracellular Ca^2+^ level (Fig. 2a, b). Furthermore, treatment of THP-1 cells with ionomycin A (Ca^2+^ ionophore) which induced [Ca^2+^]_i_ elevation and increased intracellular Ca^2+^ concentration (Fig. 2, b) induced Aβ1-42-evoked response (decreasing the level of MerTK protein) (Fig. 2, d). Thapsigargin, an endoplasmic reticulum Ca^2+^ pump inhibitor, also induced an increase of intracellular Ca^2+^ level, mimicking Aβ1-42-evoked effects (Fig. 2a–d). However, the Aβ1-42-evoked response was significantly attenuated by depletion of Ca^2+^ with ethylene glycol tetraacetic acid (EGTA) (Fig. 2e, f). Taken together, these findings suggest that increased intracellular Ca^2+^ is required for decreased MerTK expression in response to Aβ1-42 in human THP-1 macrophages.

### Aβ1-42-mediated decrease of MerTK protein level is associated with Ca^2+^-dependent hypersecretion of IL-1β and TNF-α that could negatively regulate MerTK expression

**Fig. 3:**
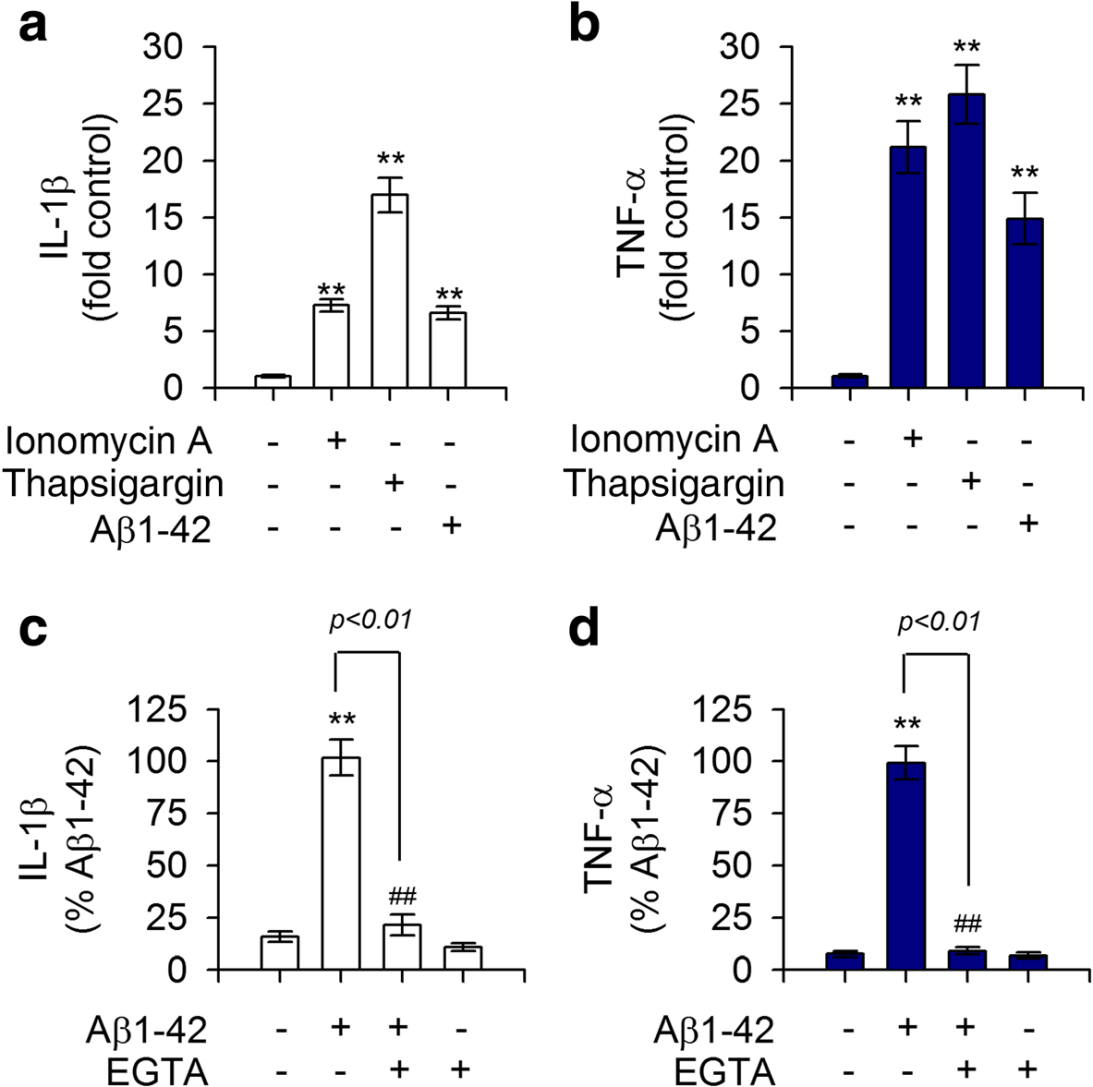
Ionomycin A or thapsigargin induced excessive production of proinflammatory cytokines IL-1β and TNF-α in comparison with treatment with Aβ1-42. **a**, **b** THP-1 cells were incubated with either the vehicle only (−), ionomycin A (4 μM), thapsigargin (2 μM), or Aβ1-42 (10 μM) for 16 h. **c**, **d** THP-1 cells were pretreated with EGTA (0.5 mM) for 30 min followed by incubation with either the vehicle only (−) or Aβ1-42 (10 μM) for 16 h. Levels of IL-1β (**a**, **c**) or TNF-α (**b**, **d**) in supernatants were quantitated by ELISA. Ionomycin A and thapsigargin mimicked Aβ1-42-mediated effects, increasing the production and release of IL-1β and TNF-α whereas depletion of Ca^2+^ with EGTA attenuated Aβ1-42-evoked responses. Values are expressed as means ± SEM of at least six independent experiments measured in duplicates. **P < 0.01, versus vehicle-treated samples; ^##^*P* < 0.01, compared to Aβ1-42-treated samples. Aβ, amyloid-β; EGTA, ethylene glycol trtraacetic acid; IL-1β, interleukin 1-β; TNF-α, tumor necrosis factor-α

**Fig. 4:**
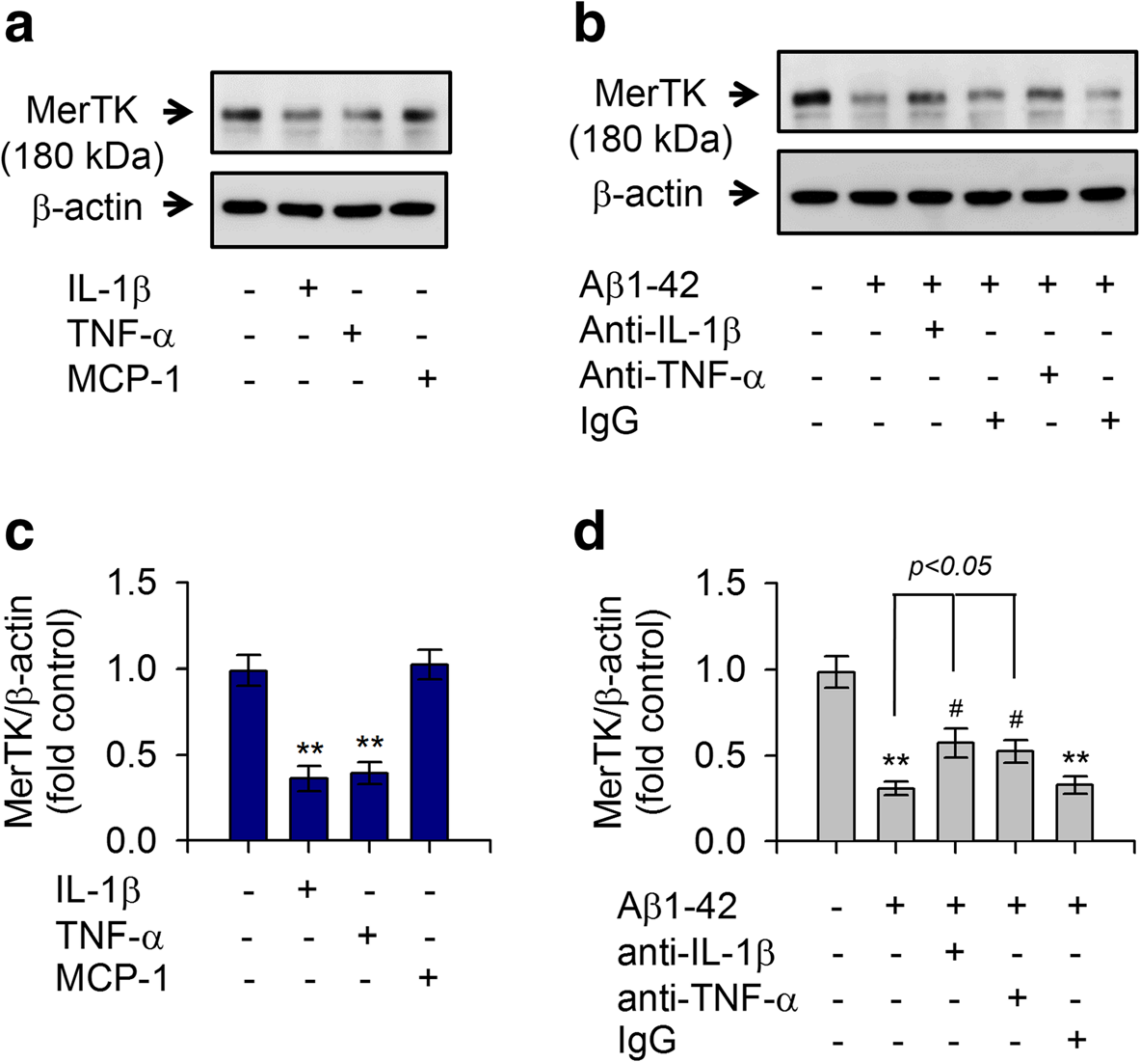
IL-1β and TNF-α decreased MerTK expression whereas neutralizing antibodies against IL-1β and TNF-α inhibited Aβ1-42-mediated reduction in MerTK expression. **a** THP-1 cells were incubated with either the vehicle only (−), IL-1β, TNF-α, or MCP-1 (10 ng/mL each) for 8 h. **b** THP-1 cells were exposed to Aβ1-42 (10 μM) for 8 h in the presence or absence of neutralizing anti-IL-1β, anti-TNF-α antibodies, or preimmune IgG (0.1 μg/mL each). Total cell lysates were examined for MerTK protein via immunoblot as described in Fig. 1. Results are representative of three independent experiments. **c**, **d** Densitometric quantification of analyses of **a** and **b** showing levels of MerTK protein. All data are presented as means ± SEM (*n* = 3–5). **P < 0.01, versus vehicle-treated samples; ^#^*P* < 0.05, compared to Aβ1-42-treated samples. Aβ, amyloid-β; IgG, immunoglobulin G; IL-1β, interleukin 1-β; MCP-1, monocyte chemoattractant protein-1; MerTK, Mer tyrosine kinase; TNF-α, tumor necrosis factor-α

Expression of MerTK is prominently upregulated in immunosuppressive environments whereas Axl levels are increased by proinflammatory factors [24]. Thus, we measured the production of proinflammatory cytokines upon stimulation with Aβ1-42 in human THP-1 macrophages. Expression levels of TNF-α and IL-1β were greatly increased in response to treatment with Aβ1-42 in THP-1 macrophages (Fig. 3a, b). Notably, levels of these cytokines were also increased in THP-1 cells treated with either ionomycin A or thapsigargin (Fig. 3, b). In contrast, the Aβ1-42-mediated increase of IL-1β and TNF-α was attenuated by depletion of Ca^2+^ with EGTA (Fig. 3c, d). To further examine the relationship between the marked decrease in MerTK protein level and the excessive production of these cytokines, THP-1 cells were exposed to recombinant TNF-α or IL-1β. As shown in Fig. 4, both TNF-α and IL-1β potently decreased MerTK protein levels whereas MCP-1 did not alter MerTK protein expression (Fig. 4a, c). Moreover, neutralizing antibodies against IL-1β or TNF-α significantly inhibited the Aβ1-42-mediated reduction of MerTK protein level (Fig. 4b, d). These observations indicate that Aβ1-42 treatment induced a decrease of MerTK protein level via increasing intracellular Ca^2+^ levels as shown in Figs. 2 and 3, consequently resulting in excessive secretion of proinflammatory cytokines such as IL-1β and TNF-α, which could act as negative feedback regulators of MerTK expression in human THP-1 macrophages.

### Sulforaphane attenuates Aβ1-42-induced MerTK reduction through inhibiting intracellular Ca^2+^ overload and NF-κB signaling

**Fig. 5:**
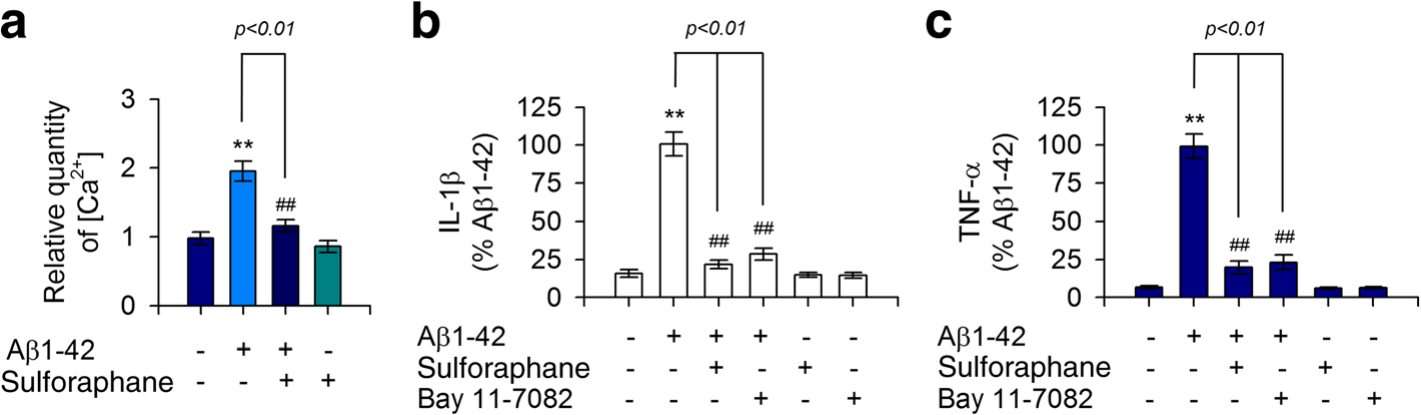
Sulforaphane decreased intracellular Ca^2+^ levels and the production of IL-1β and TNF-α provoked by Aβ1-42 insult. **a** THP-1 cells were pretreated with sulforaphane (5 μM) for 30 min and then exposed to Aβ1-42 for 8 h. Intracellular Ca^2+^ levels were measured as described in Fig. 2. **b**, **c** THP-1 cells were pretreated with sulforaphane (5 μM) for 30 min and then exposed to Aβ1-42 for 16 h. Levels of IL-1β (**b**) or TNF-α (**c**) in the supernatants were quantitated by ELISA. Values are expressed as means ± SEM of at least six independent experiments measured in duplicates. **P < 0.01, versus vehicle-treated samples; ^##^*P* < 0.01, compared to Aβ1-42-treated samples. Aβ, amyloid-β; IL-1β, interleukin 1-β; TNF-α, tumor necrosis factor-α

**Fig. 6:**
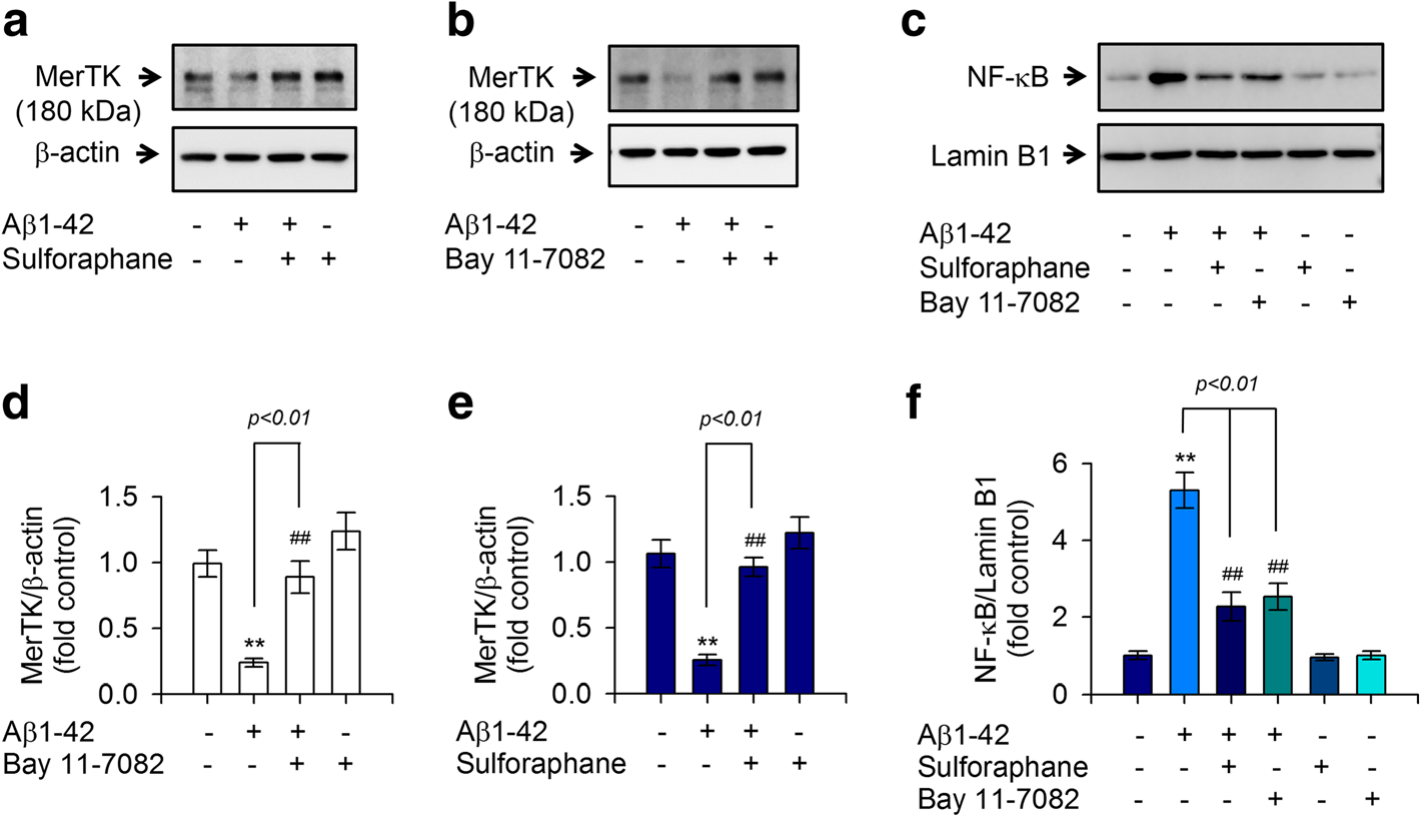
Sulforaphane rescued an Aβ1-42-mediated decrease of MerTK expression through inhibiting NF-κB nuclear translocation. **a**, **b** THP-1 cells were pretreated with **a** sulforaphane (5 μM) or **b** BAY 11-7082 (20 μM) for 30 min and then exposed to Aβ1-42 for 8 h. MerTK protein in cell lysates was measured as described above. **c** THP-1 cells were pretreated with sulforaphane (5 μM) or BAY 11-7082 (20 μM) for 30 min and then exposed to Aβ1-42 for 8 h. NF-κB protein in nuclear extracts was measured by Western blotting and normalized against the level of lamin B1. **d**–**f** Densitometric analysis of **a**–**c**. Values are expressed as means ± SEM of triplicate experiments. **P < 0.01, compared to vehicle-treated samples; ^##^*P* < 0.01, compared to Aβ1-42-treated samples. Aβ, amyloid-β; MerTK, Mer tyrosine kinas; NF-κB, nuclear factor kappa B

Despite recent growing evidence suggesting the beneficial effect of sulforaphane on Aβ pathology of AD [16, 18, 25, 26], the effect of sulforaphane on MerTK expression upon Aβ1-42 stimulation and the underlying signaling pathway remain unclear. Thus, we determined whether sulforaphane could decrease the production of these proinflammatory cytokines and attenuate Aβ1-42-mediated reduction of MerTK protein level under the same experimental paradigm. Results showed that sulforaphane potently inhibited intracellular Ca^2+^ levels (Fig. 5a) and the reduction of MerTK expression was provoked by Aβ1-42 stimulation (Fig. 6a, d). Furthermore, sulforaphane significantly reduced the hypersecretion of both IL-1β and TNF-α in Aβ1-42-treated THP-1 macrophages (Fig. 5b, c). Subsequent mechanistic studies demonstrated that application of sulforaphane resulted in a significant decrease in nuclear translocation of NF-κB induced by Aβ1-42 insult (Fig. 6c, f). Consistently, BAY 11-7082, a pharmacological NF-κB inhibitor mimicking the effect of sulforaphane mentioned earlier, reduced the nuclear translocation of NF-κB (Fig. 6c, f) and decreased the reduction of MerTK protein level in Aβ1-42-treated cells (Fig. 6b, e), thereby inhibiting the production of both IL-1β and TNF-α (Fig. 5b, c). These results confirmed that sulforaphane could inhibit NF-κB signaling and restore MerTK levels, ultimately decreasing IL-1β and TNF-α production in human THP-1 macrophages exposed to Aβ1-42.

### MerTK siRNA reduces anti-inflammatory activities of sulforaphane against Aβ1-42-induced innate immune response

**Fig. 7:**
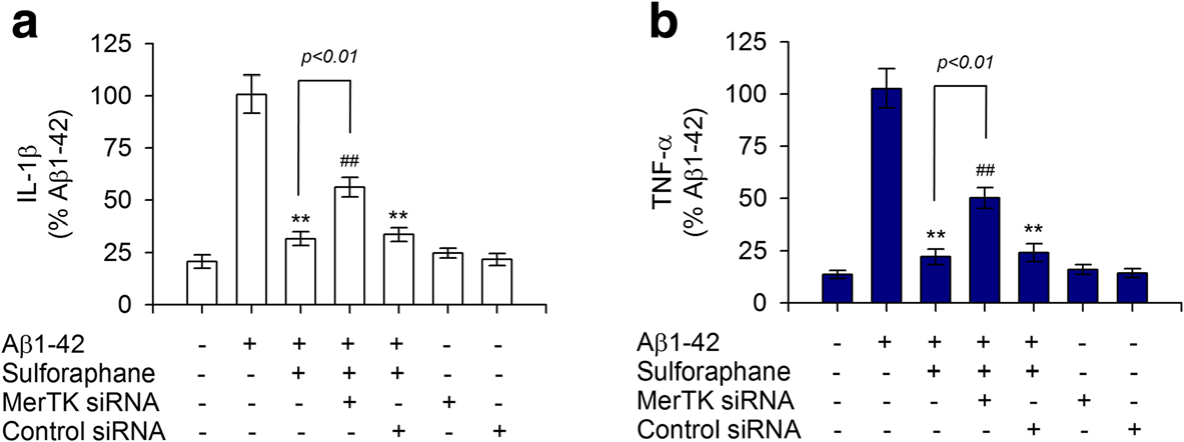
MerTK silencing using siRNA decreased anti-inflammatory activities of sulforaphane against Aβ1-42-induced production of IL-1β and TNF-α. **a**, **b** THP-1 cells were transfected with 100 ng/mL MerTK siRNA or control siRNA for 16 h. Transfected THP-1 cells were then exposed to Aβ1-42 (10 μM) for 16 h after pretreatment with sulforaphane (5 μM) for 30 min. Levels of IL-1β (**a**) or TNF-α (**b**) in culture media were measured as described above. Values are expressed as means ± SEM of at least three independent experiments measured in duplicates. **P < 0.01, compared to Aβ1-42-treated samples; ^##^*P* < 0.01, compared to sulforaphane-treated samples. Aβ, amyloid-β; IL-1β, interleukin 1-β; TNF-α, tumor necrosis factor-α; siRNA, small interfering RNA

Recent reports have suggested that depletion of MerTK which is indispensable for negative regulation of innate immune response [6, 7] may contribute to chronic inflammation implicated in AD pathology [13]. Since sulforaphane could restore an Aβ1-42-induced decrease of MerTK, next, we evaluated if this increase of MerTK was responsible for sulforaphane’s anti-inflammatory activities against excessive production of IL-1β or TNF-α upon Aβ1-42 stimulation. As shown in Fig. 7, depletion of MerTK with siRNA significantly diminished sulforaphane’s anti-inflammatory effects on Aβ1-42-induced production of proinflammatory cytokines, thus increasing the release of IL-1β and TNF-α. However, control siRNA had little effect (Fig. 7a, b). These results demonstrated that depletion of MerTK could significantly relieve sulforaphane’s anti-inflammatory activity against Aβ1-42 and consequently restore Aβ1-42-elicited production of proinflammatory cytokines such as IL-1β and TNF-α in human THP-1 macrophages.

## Discussion

Results of the present study indicated that Aβ1-42 elicited a significant decrease in MerTK expression accompanied by increased intracellular Ca^2+^ level, thereby overproducing IL-1β and TNF-α in human THP-1 macrophages. These effects were mimicked by ionomycin A or thapsigargin and attenuated by depletion of Ca^2+^ with EGTA. Interestingly, recombinant IL-1β or TNF-α potently decreased MerTK expression whereas immunodepletion of IL-1β or TNF-α with neutralizing antibodies significantly rescued the decrease in MerTK protein level provoked by Aβ1-42 treatment, implicating a negative feedback regulation of MerTK expression by IL-1β and TNF-α. Notably, sulforaphane inhibited Aβ1-42-induced downregulation of MerTK expression by decreasing the overload of intracellular Ca^2+^ level, ultimately inhibiting IL-1β and TNF-α production. A subsequent mechanistic study revealed that inhibition of NF-κB signaling was involved in the anti-inflammatory effect of sulforaphane as BAY 11-7082, a NF-κB inhibitor, mimicked the aforementioned effect of sulforaphane. Moreover, siRNA-mediated knockdown of MerTK diminished the anti-inflammatory effect of sulforaphane against Aβ1-42, consequently resulting in increased production of IL-1β and TNF-α. These findings suggest that MerTK is indispensable for the negative regulation of innate immune response provoked by Aβ1-42 in human macrophages. Moreover, the anti-inflammatory potential of sulforaphane through inhibiting an Aβ1-42-mediated decrease in MerTK protein level strongly supports the notion that sulforaphane may serve as a potential nutraceutical agent for AD.

Results from the present study clearly provide direct evidences that negative modulation of MerTK expression by Aβ1-42 occurs through increased intracellular Ca^2+^ levels, subsequently leading to hypersecretion of IL-1β and TNF-α in human THP-1 macrophages. First, ionomycin A (Ca^2+^ ionophore) and thapsigargin (an endoplasmic reticulum Ca^2+^ pump inhibitor) increase the intracellular Ca^2+^ level and decrease the level of MerTK protein while they increase the production of IL-1β and TNF-α. Second, these Aβ1-42-evoked responses were attenuated by depletion of Ca^2+^ with EGTA. These observations indicate that an increase of intracellular Ca^2+^ levels resulted in a decrease of MerTK protein level which ultimately released excessive IL-1β and TNF-α upon exposure to Aβ1-42. Thus, this is the first report showing that Aβ1-42 can decrease MerTK expression through intracellular Ca^2+^ overload, leading to the excessive production of IL-1β and TNF-α in human macrophages. Interestingly, recombinant IL-1β or TNF-α treatment elicited a decrease in MerTK protein level. Furthermore, immunodepletion of IL-1β or TNF-α with neutralizing antibodies significantly inhibited an Aβ1-42-mediated decrease in MerTK protein level. These findings implicate a negative feedback regulation of MerTK expression by IL-1β and TNF-α in human THP-1 macrophages. Our observation is consistent at least in part with a previous report demonstrating that MerTK is induced in immunosuppressive environments whereas Axl levels are increased by proinflammatory factors [24]. Interestingly, a more recent study has demonstrated that MerTK is downregulated in classically activated microglial cells while it is one of the proteins mostly upregulated in TGF-β-treated microglia cells [27].

Both MerTK and Axl receptors as markers of alternative activation for anti-inflammatory actions in macrophages can act as negative feedback regulators to reduce inflammation. They are responsible for promoting tissue repair and phagocytosis in tissue macrophages [6, 7, 28]. In particular, roles for MerTK in mediating phagocytosis and clearance of apoptotic cells have been described in MerTK−/− macrophages [29] via tethering apoptotic cells to macrophage surface and driving their subsequent internalization [30]. Nevertheless, a previous study has demonstrated that abundant inflammatory macrophages are principally associated with extracellular deposits of amyloid in the AD brain. These macrophages are unable to efficiently phagocytose or clear plaques from the brain [2]. More studies have also reported that the interaction of macrophages with plaques elicits the secretion of an array of inflammatory cytokines that can directly suppress phagocytosis [31]. In addition, the presence of amyloid acts to suppress macrophage phagocytic function [32]. In this regard, our results clearly indicate that the interaction of infiltrating macrophages upon exposure to amyloid could directly suppress MerTK protein level, which can permit excessive secretion of proinflammatory cytokines such as IL-1β and TNF-α, consequently resulting in phagocytically inactive macrophages. Furthermore, our data demonstrating a negative feedback regulation of the MerTK expression by IL-1β and TNF-α excessively induced upon Aβ1-42 exposure might explain why inflammatory macrophages fail to effectively phagocytize amyloid deposits, leading to progressive accumulation of plaques. Consistently, a recent study has demonstrated that induction of phagocytic receptor MerTK can enhance phagocytic capacity and reduce plaque burden in murine models of AD [13], strongly supporting results of our study. It is also important to note that MerTK plays a critical role in adult neurogenesis [12]. In addition, adult mice deficient in microglial MerTK have exhibited marked accumulation of apoptotic cells in neurogenic regions of the CNS [9].

This is also the first report to demonstrate that sulforaphane can restore the depletion of MerTK and inhibit IL-1β and TNF-α production through decreasing intracellular Ca^2+^ overload and attenuating NF-κB activation in human THP-1 macrophages exposed to Aβ1-42. A decrease in intracellular Ca^2+^ level caused by sulforaphane treatment is consistent with a recent study demonstrating that sulforaphane could inhibit Ca^2+^ overloading induced by methylmercury [33]. Furthermore, BAY 11-7082, a NF-κB inhibitor, restored the depletion of MerTK and decreased the excessive secretion of IL-1β and TNF-α through attenuating NF-κB signaling in Aβ1-42-stimulated human THP-1 macrophages, mimicking the effect of sulforaphane. Taken together, these data provide direct evidence that the anti-inflammatory mechanism of sulforaphane involved in upregulating MerTK expression is through inhibiting Ca^2+^ overloading and attenuating NF-κB signaling in human macrophages exposed to Aβ1-42, at least in part. Results of this study implicate that phytochemical sulforaphane might be used as a preventive and/or therapeutic target for AD management in addition to recently growing evidence suggesting beneficial effects of sulforaphane on Aβ pathology of AD [16, 18, 25, 26].

It has been reported that sulforaphane can suppress lipopolysaccharide (LPS)-induced IL-1β secretion via inhibiting transcriptional activity of NF-κB [34]. In addition, sulforaphane can suppress LPS-induced inflammation in mouse peritoneal macrophages through NRF2-dependent pathway [35]. Our recent study has also shown that sulforaphane possesses anti-inflammatory effects against Aβ1-42 through STAT-1 dephosphorylation and activation of NRF2/HO-1 cascade in human THP-1 macrophages [18]. These findings together imply that the anti-inflammatory effect of sulforaphane is likely due to its activities against various molecular targets, some of which might be interdependent. Therefore, more work is required to elucidate the mechanisms underlying the intricate signaling crosstalk between NF-κB inactivation and NRF2/HO-1 activation. Importantly, anti-inflammatory activities of sulforaphane at a multitude of molecular targets would be beneficial to the exploitation of multitarget-directed drugs to control AD [36], given that AD is a multifactorial disease, of which approximately 95% occurs sporadically with unknown origin while 5% may be due to familial AD [37].

Notably, our finding that depletion of MerTK with siRNA significantly suppressed sulforaphane’s anti-inflammatory activities against Aβ1-42 clearly implicates that MerTK plays a pivotal role in the negative regulation of innate immune response. Our recent study has demonstrated that inhibiting MerTK kinase can enhance inflammatory responses in LPS-induced acute lung injury [38]. We have also observed that restoring MerTK protein expression by treatment with TNF-α processing inhibitor-0 (TAPI-0) can efficiently prevent the inflammatory cascade during acute lung injury [39]. Moreover, an earlier study has shown that a lack of Mer inhibitory signal in *mer*KD mice will lead to elevated and prolonged NF-κB activation, causing excess macrophage activation and TNF-α production [40]. Taken together, these observations suggest that MerTK is indispensable for the negative regulation of innate immune response provoked by Aβ1-42. In particular, MerTK depletion could contribute to a decrease of Aβ phagocytic activity [13], increasing Aβ accumulation and chronic inflammation implicated in AD pathology. Further study remains to present the Aβ phagocytosis following MerTK depletion in the macrophages/microglia with sulforaphane to provide important molecular insights into AD and sulforaphane’s therapeutic potential.

## Conclusion

**Fig. 8:**
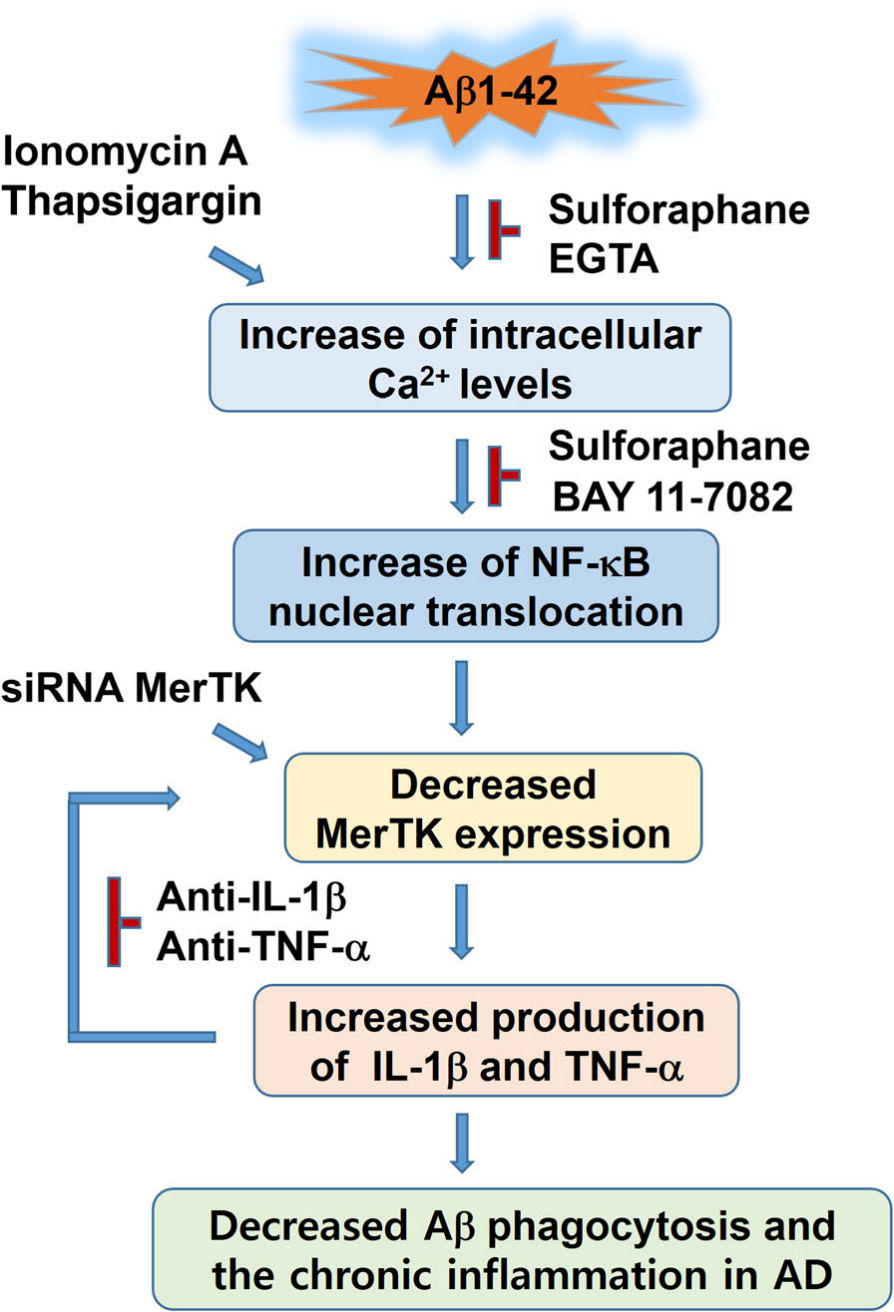
Proposed mechanisms for beneficial effects of sulforaphane against decreased MerTK expression following Aβ1-42 insult in human THP-1 macrophages. Sulforaphane attenuates Aβ1-42-induced MerTK reduction through inhibiting intracellular Ca^2+^ overload and NF-κB signaling as replicated by EGTA and BAY 11-7082. Consequently, sulforaphane rescues a decrease of MerTK expression following Aβ1-42 stimulation, thereby inhibiting overproduction of IL-1β and TNF-α. Interestingly, Αβ1-42-induced IL-1β and TNF-α could act as negative feedback regulators of MerTK expression as confirmed with neutralizing antibodies against IL-1β or TNF-α, implicating that MerTK downregulation and induction of IL-1β and TNF-α by Aβ1-42 stimulation are interdependent. Depletion of MerTK with siRNA significantly suppressed the sulforaphane’s anti-inflammatory activities against Aβ1-42, implicating a pivotal role of MerTK for the negative regulation of the innate immune response elicited by Aβ1-42 in human THP-1 macrophages

The present study clearly demonstrated that Aβ1-42 could increase in intracellular Ca^2+^ level and NF-κB activation which potently decreased MerTK expression in human THP-1 macrophages, consequently overproducing IL-1β and TNF-α. Furthermore, Aβ1-42-induced IL-1β and TNF-α could decrease MerTK protein level, implicating a negative feedback regulation of MerTK expression by IL-1β and TNF-α as summarized in Fig. 8. Notably, sulforaphane significantly rescued an Aβ1-42-mediated decrease of MerTK protein level through mechanisms involving decreases of Ca^2+^ overloading and NF-κB activation, thereby inhibiting the excessive production of IL-1β and TNF-α, at least in part. Therefore, these findings together implicate that targeting of MerTK with phytochemical sulforaphane as a mechanism for preventing Aβ1-42-induced neuroinflammation might have therapeutic potential for AD management, although the relevance of these in vivo findings remains to be clearly elucidated.

## Abbreviations

Aβ: Amyloid-β
ActD: Actinomycin D
AD: Alzheimer’s disease
CHX: Cycloheximide
CNS: Central nervous system
EGTA: Ethylene glycol tetraacetic acid
ELISA: Enzyme-linked immunosorbent assay
IgG: Immunoglobulin G
IL-1β: Interleukin 1β
MCP-1: Monocyte chemoattractant protein-1
MerTK: Mer tyrosine kinase
NF-κB: Nuclear factor kappa B
NRF2: Nuclear factor erythroid 2-related factor 2
RTKs: Receptor tyrosine kinases
SDS-PAGE: Sodium dodecyl sulfate polyacrylamide gel electrophoresis
SEM: Standard error of the mean
siRNA: Small interfering RNA
STAT-1: Signal transducer and activator of transcription-1
TAPI-0: TNF-α processing inhibitor-0
TNF-α: Tumor necrosis factor-α
